# Tissue Oxidative Ecology along an Aridity Gradient in a Mammalian Subterranean Species

**DOI:** 10.3390/antiox11112290

**Published:** 2022-11-18

**Authors:** Paul J. Jacobs, Daniel W. Hart, Hana N. Merchant, Andries K. Janse van Vuuren, Chris G. Faulkes, Steven J. Portugal, Barry Van Jaarsveld, Nigel C. Bennett

**Affiliations:** 1 Department of Zoology and Entomology, Mammal Research Institute, University of Pretoria, Pretoria 0002, South Africa; 2 Department of Biological Sciences, School of Life and Environmental Sciences, Royal Holloway University of London, Egham, Surrey TW20 0EX, UK; 3 School of Biological and Behavioural Sciences, Queen Mary University of London, Mile End Road, London E1 4NS, UK; 4 Department of Physical Geography, Faculty of Geosciences, Utrecht University, Princetonlaan 8a, 3584 CB Utrecht, The Netherlands

**Keywords:** oxidative stress, redox balance, oxidative status, life history, survival, precipitation, sociality, aridity, water balance, mole-rat

## Abstract

Climate change has caused aridification which can alter habitat vegetation, soil and precipitation profiles potentially affecting resident species. Vegetation and soil profiles are important for subterranean mole-rats as increasing aridity causes soils to become harder and geophytes less evenly distributed, and the inter-geophyte distance increases. Mole-rats obtain all water and dietary requirements from geophytes, and thus digging in harder soils may amplify stressors (hyperthermia, dehydration- or exercise-induced damage). This study assessed the oxidative status of the wild common mole-rat along an aridity gradient (arid, semi-arid and mesic). Kidney and liver oxidative markers, including total oxidant status (TOS), total antioxidant capacity (TAC), oxidative stress index (OSI), malondialdehyde (MDA) and superoxide dismutase (SOD) were measured. Liver oxidative status did not demonstrate any significance with the degree of the aridity gradient. Aridity affected the TAC and OSI of the kidney, with individuals in the most arid habitats possessing the highest TAC. The evolution of increased group size to promote survival in African mole-rats in arid habitats may have resulted in the additional benefit of reduced oxidative stress in the kidneys. The SOD activity of the kidneys was higher than that of the liver with lower oxidative damage, suggesting this species pre-emptively protects its kidneys as these are important for water balance and retention.

## 1. Introduction

Animals have evolved specific behavioural, morphological, physiological and molecular adaptations to aid survival in their respective habitats [1,2,3,4]. Of late, organism adaptations to aridity (a region with low and unpredictable precipitation often accompanied by extreme temperatures) have become a focal point of research [5]. The aridity index (AI) indicates how wet or dry a region is; the lower the AI, the drier or less wet the region [5,6]. Aridification is the process of the AI declining; for example, a mesic region (a region with a moderate or well-balanced supply of water and a moderate temperature range) can become semi-arid through the process of aridification, changing temperature and precipitation profiles [5,6]. Consequences of aridification, as the result of climate change, may include changes to a habitat’s soil drought profile, overall water availability [6,7,8,9,10], vegetation types and floristic biodiversity [11,12,13,14]. Understanding adaptations of organisms in different habitat aridities could be pivotal to our understanding of the possible changes required by organisms towards future aridification and climate extremes.

Animals that may be sensitive to changes in aridity are subterranean rodents, where a significant contributor to their increased sensitivity is the energetic costs associated with digging and the corresponding heat generation [15,16,17]. Excavation of tunnel systems is energetically more expensive (up to 3.6 times) than aboveground exploration [15,16]. Aridification may force subterranean rodents to dig in harder soils more frequently, which can result in a detrimental elevation of core body temperatures [17,18], with the possibility of exercise-induced hyperthermia [19,20]. Furthermore, aridification may affect the foraging of subterranean rodents; food sources would become more clumped and dispersed due to changes in flora, while foraging would become difficult due to increased soil hardness [21].

Oxidative stress has previously been used as a measure of the consequences of heat stress and/or dehydration [22,23,24,25,26] and exercise-induced damage [19,20,27,28,29,30,31]. Oxidative stress occurs when the oxidative balance is compromised in favour of reactive oxygen species (ROS) at the expense of antioxidant activity, which cannot prevent the overproduction of free radicals [32,33,34,35,36]. Additionally, oxidative stress can bring about physiological changes to lipids, DNA and proteins, which can compromise or disrupt cellular function and signalling [34,35,37,38]. Although physiological parameters have been investigated along an aridity gradient in mammals [39,40,41,42,43,44], to date, no study has investigated the oxidative status parameters of a mammal across an aridity gradient. The family Bathyergidae occupies a wide range of habitats from mesic to hyper-arid [45,46,47], as well as displaying a unique cline of sociality ranging from solitary, social and some species considered eusocial [48,49,50]. As such, this study set out to determine the variation in oxidative status of a single subterranean mammalian species, the common mole-rat (*Cryptomys hottentotus hottentotus*), across an aridity gradient, where this species occupies arid, semi-arid and mesic habitats [21]. A single species was chosen to avoid species-specific variation in oxidative status.

The ecological constraints and foraging risks associated with digging in harder soils in arid environments were proposed as conditions for mole-rat social behaviour termed the arid food distribution hypothesis [21,51]. Social behaviour would mitigate the energy expenditure required for digging in harder soils and allow groups to be more efficient in obtaining all their nutrient and water requirements from the storage organs of underground geophytes [21,51]. Thus, group living would alleviate the effects of aridification, where a group of mole-rats would be better equipped to deal with harsher environments [21,51]. Despite the benefit of group living, the subterranean niche has disadvantages, including hypoxic and hypercapnic conditions with poor ventilation and high humidity, which is exacerbated by increased group sizes [52,53,54,55,56]. The thermal environment of the burrow is fairly uniform, and temperatures are muted depending on soil depth, resulting in a limited preference for a thermal environment to potentially dissipate heat [57]. This suggests heat generation during digging is unlikely to be offloaded readily, resulting in possible hyperthermic changes in a subterranean animal when digging [16].

African mole-rats can utilise behavioural and physiological means of mitigating the harsh effects of an arid environment [48,49,57,58,59,60]. Firstly, a regular food resource is particularly important, as African mole-rats do not drink free-standing water but obtain all of their water requirements from the underground geophytes upon which they feed [61,62,63]. It was proposed by Hart et al. [60] that group living in mole-rats allows for increased efficiency in foraging for food, which culminates in year-round water availability, effectively resulting in behavioural osmoregulation. Mole-rats can readily use this excess water to urinate on themselves to increase evaporative cooling [59]. This is supported by a very high-water economy index (units of mL water used per kJ energy metabolized) in Damaraland mole-rats (*Fukomys damarensis*) despite inhabiting an arid environment [60]. African mole-rats can also selectively forage during post-rainfall periods, as moist soil is preferable to work with [64,65]. This allows digging in cooler soils to result in heat transfer via conduction, possibly allowing the mole-rat to dig more intensely and for longer periods [66]. Additionally, mole-rats can limit activity times during cooler periods regardless of rainfall, where heat stress may be less impactful [59,67,68]. Physiological adjustments include a higher conductance than rodents of comparable body sizes, which can assist in additional energy and water saving, as well as not requiring investment in energetically costly cooling mechanisms [48,57,59,69,70]. Additionally, mole-rats do not typically lower their metabolic rate due to living in an arid environment as surface-dwelling rodents do, as no correlation between aridity and metabolic rate occurs in mole-rats [61,71].

The current study investigated the oxidative status of the liver and kidney of the common mole-rat along an aridity gradient. Although the skeletal muscle and the heart [29] are vulnerable to exercise-induced oxidative stress [72,73,74,75], the liver [20,29,76,77] and the kidney [20,78,79,80] have also demonstrated vulnerability to exercise-induced oxidative stress; importantly, however, the kidney [80,81,82,83] and liver [22,84,85] are also susceptible to hyperthermia [86] and/or dehydration stress [22], making these tissues more likely to be stressed during hyperthermia-induced oxidative stress [87]. Since the kidneys are the most important organ for water retention and osmoregulation [88,89,90] and the liver susceptibility to hyperthermia [87], and a source of glutathione, an essential thiol for free radical scavenging during exercise [91], these two tissues were chosen for the current study. Differences in oxidative status and antioxidant activity of the liver and the kidney are provided as supplementary material (Supplementary S1). Markers used to investigate oxidative stress include antioxidant activity through non-enzymatic total antioxidant activity (TAC) and enzymatic antioxidant activity through the superoxide dismutase (SOD) enzyme. We measured total oxidant status (TOS) and malondialdehyde (MDA) as measures of oxidative damage potential and lipid oxidative damage, respectively. Lastly, we used the TOS:TAC measurement as an indicator of the oxidative stress index (OSI).

In accordance with behavioural osmoregulation and the aridity food-distribution hypothesis, possible oxidative stress caused by exercise-induced hyperthermia, temperature and/or dehydration brought about by digging in arid conditions would be negated as individuals that live in a larger group size would share digging duties with their colony mates and have a constant supply of food and water. Therefore, we predict that although habitats differ in aridity, the effect of differing aridity on oxidative damage (TOS and MDA), antioxidant activity (TAC and SOD) and oxidative stress index (OSI) for each tissue (kidney and liver) will have similar concentrations. We intend to discuss the results of our findings in light of aridification post hoc.

## 2. Materials and Methods

### 2.1. Study Area, Sampling Sites and Sampling Sites’ Climate Data and Analysis

The arid site (Steinkopf) is defined as the Richtersveld bioregion. The Richtersveld is a desert landscape characterised by rugged kloofs and high mountains, situated in the north-western corner of South Africa’s Northern Cape province. The Richtersveld plant life grows in rocky surfaces, gravel slopes, sandy sheet washplains and riverbeds in nutrient-poor soils (similar to Fynbos regions). The Richtersveld plant life is diverse and mainly endemic to South Africa comprising mainly succulents and aloe species, interspersed with succulent geophytes. This area receives a winter rainfall.

The semi-arid site (Darling) is defined as the west coast Renosterveld bioregion. Renosterveld plants grow in rich soil (in comparison to Fynbos regions). Typically, Renosterveld is largely confined to fine-grained soils—mainly clays and silts. The vegetation type is dominated by a species of grey-coloured plant called the *renosterbos*. However, the typical vegetation of the Fynbos tends to occur in very low abundance in Renosterveld. The types of plants are grasses, shrubs and small trees and perennials with numerous geophytes. This area also receives winter rainfall.

The mesic site (Somerset West) is defined as the Southwest Fynbos bioregion. This area is predominantly coastal and mountainous, with a Mediterranean climate and rainy winters. Fynbos plant life grows in nutrient-poor soils. The Fynbos plant life is one of the most diverse in the world and mainly endemic to South Africa, and comprises mainly succulent and aloe species with an array of geophytes. The most conspicuous components of the flora are evergreen sclerophyllous plants, many with ericoid leaves and gracile habit, as opposed to timber forest. Several plant families are conspicuous in fynbos; the Proteaceae are prominent, with genera such as Protea, Leucospermum (the “pincushions”) and Leucadendron (the silver tree and “cone bushes”).

**Figure 1:**
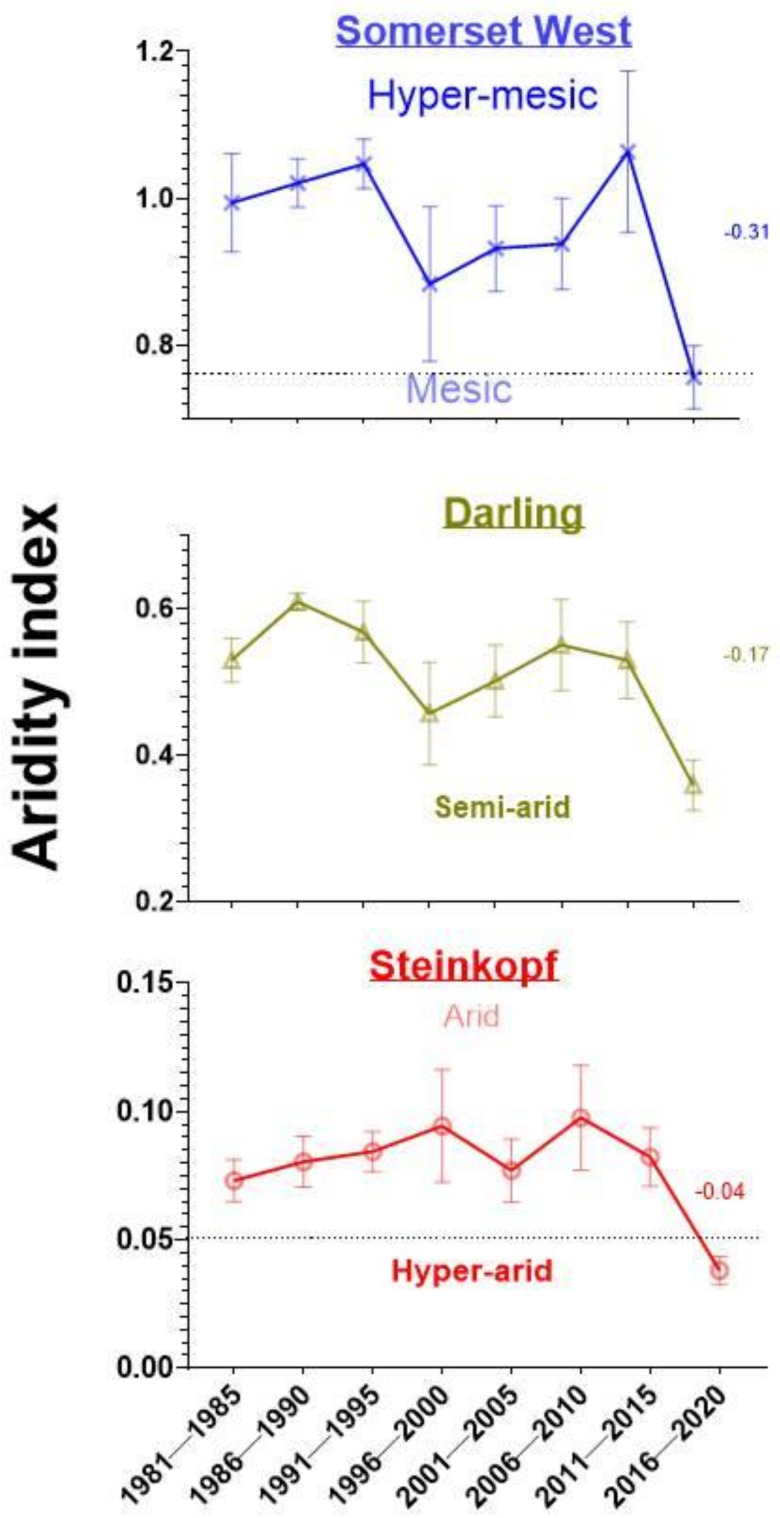
The aridity index for the last 40 years split into 5-year periods for three regions in South Africa, namely Somerset West (34.0757° S, 18.8433° E), Darling (33.3756° S, 18.3861° E) in the Western Cape and Steinkopf (29.2602° S, 17.7340° E) in the Northern Cape. Climate classification is according to Colantoni et al. [92]. Mean ± s.e.m.

According to Colantoni et al. [92], Somerset West (AI: 0.95 ± 0.03) is hyper-mesic, while Darling (AI: 0.51 ± 0.02) is semi-arid and Steinkopf (AI: 0.08 ± 0.01) is arid. Furthermore, the consequences of anthropogenic climate change within the last six years (2016-current) have exacerbated aridification, resulting in decreased AI in both the Western and Northern Cape (Figure 1). As such, Somerset West is currently classified as mesic, while Darling (AI: 0.51 ± 0.02) remains semi-arid, and Steinkopf (AI: 0.08 ± 0.01) is now hyper-arid (Figure 1).

Climate data for each site were retrieved from ERA5-Land of the European Centre for Medium-Range Weather Forecasts—the latest generation created by the Copernicus Climate Change Service [93]. The spatial resolution is 0.1 degree by 0.1 degree. Monthly averaged temperature (T_air_; °C), total precipitation (tp; m), and dew point temperature (d2m; °C) from 1981 to 2020 were used. These data were used to calculate the annual aridity index (AI) (Equation (1)), where tp was directly obtained from ERA5-Land and potential evapotranspiration (PET) was calculated from the well-known Romanenko estimation (Equation (2)) [94]. For Equation (2), relative humidity (RH) was calculated from ERA5-Land d2m (Equation (3)).

(1) $$AI=\frac{tp}{PET}$$

(2) $$PET=0.00006\times \left(100-RH\right)\times {\left(25+Tair\right)}^{2}$$

(3) $$RH=100\times 10^{7.591386\;\left(\frac{d2m}{d2m+240.763}-\frac{Tair}{Tair+240.7263}\right)}$$

### 2.2. Animal Capture

The common mole-rats were captured from three localities in South Africa, namely Somerset West (34.0757° S, 18.8433° E; Western Cape), Darling (33.3756° S, 18.3861° E; Western Cape) and Steinkopf (29.2602° S, 17.7340° E; Northern Cape) between November 2021 and March 2022. Animals were captured using Hickman live traps [95] baited with sweet potatoes. Social African mole-rats show an extreme reproductive division of labour, whereby only one female and one to three males breed. At the same time, the remaining colony members are reproductively suppressed and would not have achieved sexual maturity or participated in breeding whilst in the confines of their natal colony [49]. All animals used in this study were considered adults and reproductively inactive (non-breeders) as a consequence of reproductive suppression [96,97]. Therefore, only male and female non-breeders were used in this study (see Hart, et al. [98] on how reproductive status was determined). The use of non-breeders only circumvents complications of oxidative stress associated with reproduction [99,100].

### 2.3. Animal Housing

Animals were transported back to the laboratory at the Department of Zoology and Entomology at the University of Pretoria (25.7545° S, 28.2314° E), South Africa, where the mole-rats were then housed. At the laboratory, the mole-rats were placed in a climate-controlled room, with a temperature of 25 °C and a humidity between 40–60%. All individuals were housed separately in large polyurethane containers (70 cm × 34 cm × 54 cm) containing wood shavings and paper towelling for nesting material. The animals were fed daily with sweet potatoes and apple ad libitum.

### 2.4. Age

Relative age was determined using tooth eruption and wear as outlined by Bennett, et al. [101], where animals were placed in relative age classes. Age has been observed to affect oxidative stress [102,103,104,105]; therefore, relative age was included in all analyses.

### 2.5. Reagents

Unless otherwise stated, all chemicals, tools and reagents in this study were obtained from Merck (Pty) Ltd. (Gauteng, South Africa).

### 2.6. Euthanasia and Tissue Excision

Samples were collected from 10 individuals (all non-breeders) per site (Somerset West: 6 males and 4 females; Darling: 5 males and 5 females; Steinkopf: 4 males and 6 females). All mole-rats came from at least five or more colonies from each site. All individuals were in captivity for no longer than a month (22–28 days) prior to being euthanised with an overdose of isoflurane. All animals were weighed immediately after death (Somerset West: 57.5 ± 3.33 g; Darling: 58.3 ± 4.31 g; Steinkopf: 74.7 ± 6.58 g). All liver and kidney samples were collected at the same time of the day (10:00–13:00) to prevent daily rhythm effects. Furthermore, tissues were collected in the same order (liver then kidney) within 10 min post-mortem, with an approximate 1-min interval between tissues. This was done to prevent and/or minimise proteins and metabolites from denaturing following dissection. The liver and kidney were then washed in 20 mM phosphate-buffered saline (PBS) to remove blood and flash-frozen in liquid nitrogen, and subsequently stored at −80 °C until analysis.

### 2.7. Tissue Homogenization Procedure

Liver and kidney were homogenised on ice by 10% weight per volume of 20 mM ice-cold PBS buffer in an 8 mL Potter–Elvehjem tissue grinder. Homogenates were split into separate tubes and centrifuged according to kit specifications. The supernatant was removed and stored in a −80 °C freezer until the time of analysis.

### 2.8. TOS

Tissue supernatant TOS levels were measured through Erel’s method [106]. Briefly, this method is based on the oxidation of ferrous ion to ferric ion in the presence of various oxidative species. The oxidation reaction is enhanced by glycerol molecules, which are abundantly present in the reaction medium. The ferric ion makes a coloured complex with xylenol orange in an acidic medium. The colour intensity, measured spectrophotometrically, is related to the total amount of oxidant molecules that are present in the sample. The results are expressed in terms of micromole hydrogen peroxide equivalent per g tissue. Samples were run in duplicate and only once per plate with a repeatability of *r* = 0.89. Intra-assay variability was 4.18%.

### 2.9. MDA

The concentration of MDA was measured in all liver and kidney samples collected and was quantified using a commercially available kit (Sigma-Aldrich, cat. No. MAK085, A6283, 258105, and 360465), following standard procedures [107]. Polyunsaturated fatty acids (lipids) are susceptible to oxidative attack through ROS, resulting in MDA. The kit determines MDA content by reacting with thiobarbituric acid (TBA) to form a colorimetric complex at 532 nm. Absorbance was read using a Spectramax M2 plate reader (Molecular Devices Corp., Sunnyvale, CA, USA) and compared to a 2 mM MDA standard (2–10 nmol/mL). Results are expressed as nmol/g tissue. Samples were run in duplicate with repeatability of *r* = 1.0 and the intra-assay variability was 1.37%.

### 2.10. TAC

TAC in homogenates of liver and kidney were quantified using a commercially available kit (Antioxidant Assay Kit, Cayman Chemical Co., Ann Arbor, MI, USA) which measures the oxidation of ABTS (2.29-Azino-di- 3-ethybenzthiazoline sulphonate]) by metmyoglobin, which is inhibited by non-enzymatic antioxidants contained in the sample. Oxidized ABTS is measured by spectrophotometry at a wavelength of 750 nm. The capacity of antioxidants in the sample to inhibit oxidation of ABTS is compared with the capacity of known concentrations of Trolox. The results are expressed as µmol of Trolox equivalents per g tissue. Absorbance was read using a Spectramax M2 plate reader (Molecular Devices Corp., Sunnyvale, CA, USA). Samples were run in duplicate and only once per plate with a repeatability of *r* = 0.99. Intra-assay variability was 2.65%.

### 2.11. SOD

We measured the total SOD activity of liver and kidney samples, where SOD is an enzymatic antioxidant that catalyses the dismutation of superoxide anions to oxygen and hydrogen peroxide [25]. We measured SOD content with a commercially available kit (Superoxide Dismutase (SOD) Colorimetric Activity Kit, Arbor Assay, Arbor Assays, Ann Arbor, MI, USA) that measures the percentage of superoxide radicals that undergo dismutation in a given sample. Absorbances were read at 450 nm using a Spectramax M2 plate reader (Molecular Devices Corp., Sunnyvale, CA, USA). The results are expressed as SOD activity per milligram tissue (SOD activity/mg tissue). Samples were run in duplicate and only once per plate with a repeatability of *r* = 0.99. Intra-assay variability was 5.07%.

### 2.12. OSI

The oxidative stress index (OSI) value was calculated according to the following formula: OSI (arbitrary unit) = [(TOS, µmol H_2_O_2_ equivalent/g tissue)/(TAC, µmol Trolox equivalent/g tissue)] ∗ 100, where the mmol value in the TAC test unit was converted to µmol units as in the TOS test [108,109].

### 2.13. Statistical Analyses

All statistical analyses were performed in R 4.2.1 [110]. Tissues were analysed separately and not compared due to the discrepancy of inherent tissue differences such as cellular turnover and accumulation or repair of damage [34]. As such, tissues were not compared statistically, and only patterns between the liver and kidney were considered. The normality of the response variables TOS, TAC, OSI, MDA and SOD was determined using Shapiro–Wilk tests. The homogeneity of all dependent variables was confirmed with Levene’s test. Log transformation was used to normalise all non-normal data. Data were analysed using a linear model using the *lme4* package [111]. All initial models contained aridity; sex and aridity*sex interaction were run as predictors, with body mass and age as covariates (Table S2). Backwards elimination of linear models were performed using the *step* function of the lmerTest package in order to determine the best model for each response variable determined through the AIC criterion [112]. The best models of the backward elimination of all response variables are presented in Table 1 and Table 2, while full backward elimination of models can be viewed in the Supplementary Material (Table S2). Significant variables in the regression models were followed up with post hoc comparisons, conducted using Tukey’s HSD pairwise comparisons using the *emmeans* package [113]. Furthermore, Pearson correlations were conducted between SOD and MDA for kidney and liver from each population differing in aridity. Data are presented as mean ± standard error (s.e.m), and a *p*-value of ≤0.05 was defined as significant. Raw data can be observed in the Supplementary Material (Table S3).

## 3. Results

### 3.1. Oxidative Damage

#### 3.1.1. Total Oxidant Status (TOS)

**Figure 2:**
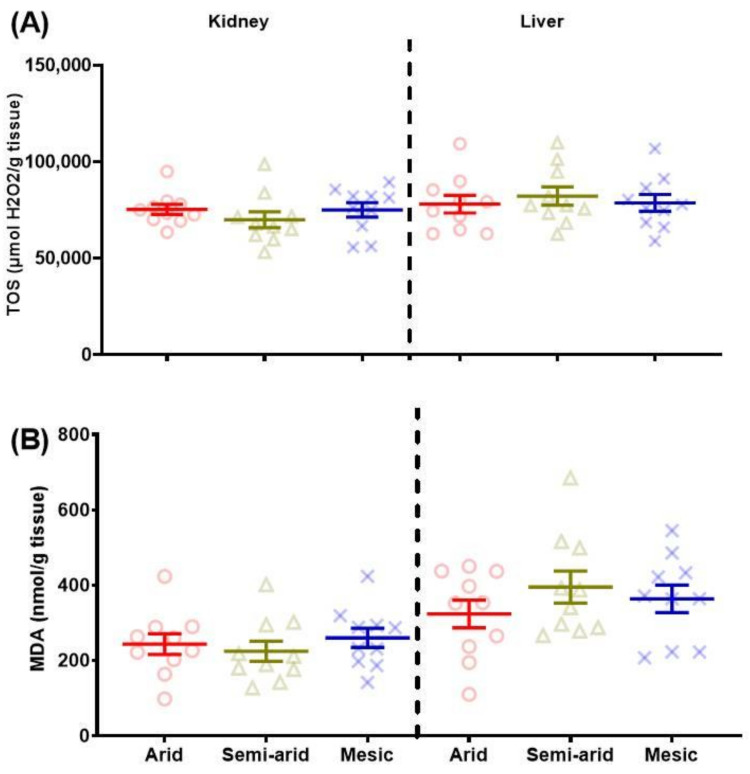
Kidney and liver (**A**) total oxidant status (TOS—μmol H_2_O_2_/g tissue) and (**B**) malondialdehyde (MDA—nmol/g tissue) of the common mole-rat (*Cryptomys hottentotus hottentotus*) for each population along an aridity gradient (arid—red (circles), semi-arid—moss (triangle), mesic—blue (x)). Mean ± s.e.m. An asterisk (*) indicates significance (*p* ≤ 0.05).

Age and body mass were not included in the kidney TOS best model (Table 1). At the same time, the best model contained aridity, sex and sex*aridity; none of these significantly affected kidney TOS levels (Table 1; Figure 2A).

The overall regression was not statistically significant, where no variable explained variation in liver TOS levels (Table 2; Figure 2A).

#### 3.1.2. Malondialdehyde (MDA)

The final regression models for kidney MDA (Table 1) and liver (Table 2) were not significant for any variable explaining variation in MDA levels (Figure 2B).

### 3.2. Antioxidant Defense

#### 3.2.1. Total Antioxidant Capacity (TAC)

**Figure 3:**
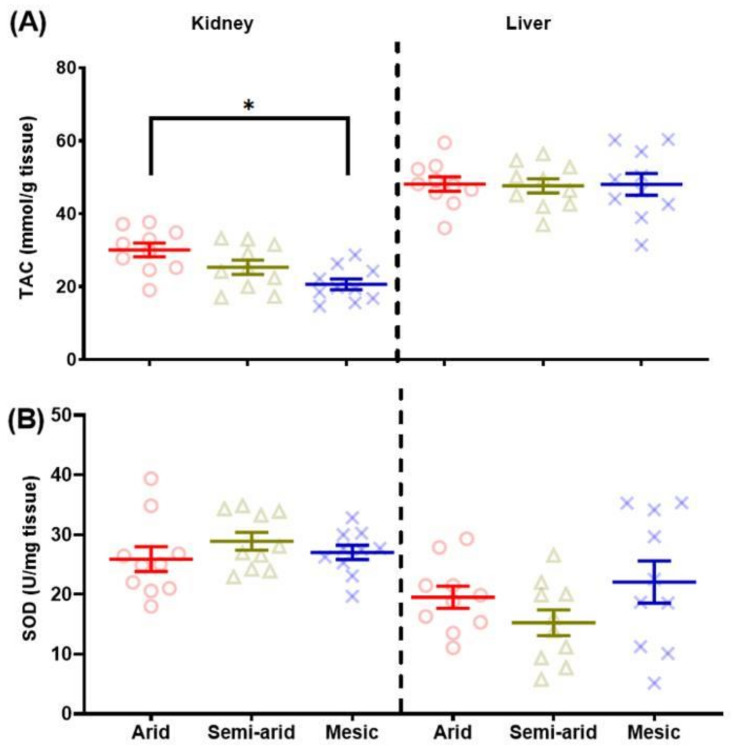
Kidney and liver (**A**) non-enzymatic total antioxidant activity (TAC—mmol/g tissue) and (**B**) enzymatic antioxidant activity through superoxide dismutase (SOD—U/mg tissue) of the common mole-rat (*Cryptomys hottentotus hottentotus*) for each population along an aridity gradient (arid—red (circles), semi-arid—moss (triangle), mesic—blue (x)). Mean ± s.e.m. An asterisk (*) indicates significance (*p* ≤ 0.05).

The overall regression was statistically significant for kidney TAC, where aridity and sex were kept in the model, with aridity being a significant contributor to the variation observed (F(_2,26_) = 7.5692, *p* = 0.003) (Table 1). Post hoc analyses for kidney TAC show that the most arid individuals had the highest TAC as compared to semi-arid (t = −2.104, *p* = 0.1086) individuals and significantly higher compared to mesic individuals, which possessed the lowest TAC (t = 4.133, *p* = 0.0009; Figure 3A). Mesic and semi-arid populations did not differ significantly from each other (t = 2.071, *p* = 0.1157) (Figure 3A).

In the final regression model, no variables explained any variation observed in liver TAC levels (Table 2; Figure 3A).

#### 3.2.2. Superoxide Dismutase (SOD)

Age was the only variable to explain any variation in kidney SOD levels and was negatively correlated with age (*r* = −0.3, *p* = 0.07), but this variation was not significant (Table 1; Figure 3B).

The best model for liver SOD levels included aridity, sex and aridity*sex, where aridity explained significant variation in the main model (t = 2.886, *p* = 0.008) (Table 2; Figure 3B). Post hoc analyses of aridity showed that no population was significantly affected by aridity (arid–mesic (t = 1.043, *p* = 0.56), semi-arid–mesic (t = 2.267, *p* = 0.08) and arid–semi-arid (t = 1.213, *p* = 0.46)) (Figure 3B).

### 3.3. Oxidative Stress: OSI

**Figure 4:**
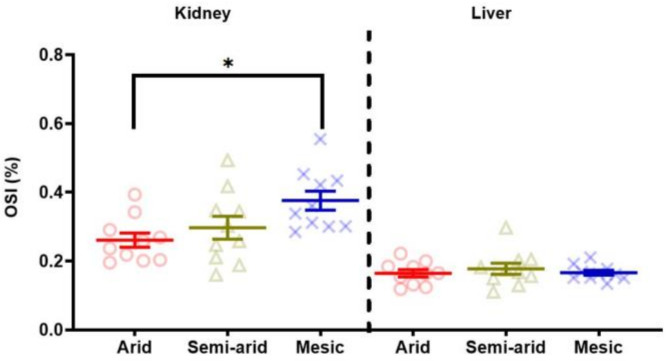
Kidney and liver oxidative stress index (OSI—%) of the common mole-rat (*Cryptomys hottentotus hottentotus*) for each population along an aridity gradient (arid—red (circles), semi-arid—moss (triangle), mesic—blue (x)). The OSI is the ratio of total oxidant status (TOS) to non-enzymatic total antioxidant activity (TAC). Mean ± s.e.m. An asterisk (*) indicates significance (*p* ≤ 0.05).

The kidney OSI regression model retained body mass, aridity and sex as variables contributing to the variation in kidney OSI, where only aridity was found to be a significant contributor to kidney OSI (F((_2,25_) = 3.9429, *p* = 0.03) (Table 1; Figure 4). Post hoc comparisons show that mesic individuals had the highest OSI compared to semi-arid individuals (t = −2.928, *p* = 0.0977) and significantly higher compared to arid individuals (t = −2.928, *p* = 0.019) (Figure 4). Conversely, arid individuals had the lowest OSI compared to semi-arid individuals, but this was not significant (t = 1.033, *p* = 0.5633) (Figure 4).

No variable explained any variation in liver OSI, with the regression model being not significant (Table 2; Figure 4).

### 3.4. SOD-MDA

**Figure 5:**
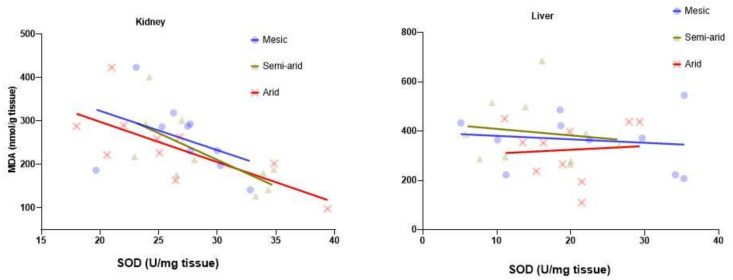
Correlations between malondialdehyde (MDA—nmol/g tissue) and superoxide dismutase (SOD—U/mg tissue) activity in the kidney and liver of the common mole-rat (*Cryptomys hottentotus hottentotus*) for each population along an aridity gradient (arid—red (circles), semi-arid—moss (triangle), mesic—blue (x)). Kidney—arid: *r* = −0.71, *p* = 0.02; semi-arid: *r* = −0.69, *p* = 0.03; mesic: (*r* = −0.42, *p* = 0.22. Liver—arid: *r* = 0.08, *p* = 0.84; semi-arid: *r* = −0.13, *p* = 0.71; mesic: *r* = −0.14, *p* = 0.71).

A negative correlation was observed between kidney SOD and MDA for all three aridity index regions, which was significant only for populations from the arid (*r* = −0.71, *p* = 0.02) and semi-arid (*r* = −0.69, *p* = 0.03) regions, but not from the mesic region (*r* = −0.42, *p* = 0.22) (Figure 5).

No significant correlation was observed between liver SOD and MDA for arid, semi or mesic regions (*r* ≤ 0.08, *p* ≥ 0.71, for all three, Figure 5).

## 4. Discussion

In this study, we investigate for the first time (while controlling for age) the oxidative ecology of a wild subterranean rodent along an aridity gradient. In contrast to our initial predictions, we found that aridity did affect oxidative stress, but this was tissue-specific and driven by both the access to food and different types of food available rather than the production of ROS or resultant oxidative damage. Although the current study did not determine group sizes between the populations, our study investigated the same populations as Spinks, Bennett and Jarvis [21], where tendencies toward larger group sizes were observed in the arid population as compared to the mesic one.

The cost of digging in an arid environment may expose mole-rats to more frequent metabolic exertion that subsequently results in increased oxidative stress, but the social lifestyle of mole-rats may prevent over-exertion during digging, preventing oxidative stress [17,66]. As predicted, TOS levels for each tissue were similar regardless of aridity, where TOS levels are an indirect measure of the total ROS production [106]. Despite the increased energetic costs and the consequent predicted increase of ROS production from increased metabolism associated with digging in drier and harder soils [15,16,17], individuals in arid environments did not appear to exhibit elevated ROS from exercise, dehydration and/or heat stress generated as a consequence of digging. This implies that individuals from the arid environment consisting of larger groups may burrow less often compared to their counterparts in the mesic populations, that would burrow more frequently due to the increased ease of digging. Support for this includes field activity rates that were higher in a mesic-dwelling social African mole-rat species, the Natal mole-rat (*C. h. natalensis*) (average colony size of seven) [68], compared to an arid-dwelling social mole-rat species, the Damaraland mole-rat (*Fukomys damarensis*) (average colony size of 16) [114]. However, Hart et al. [60] found no significant difference when comparing the daily energy expenditure of the same two species. This may be a consequence of exposure to different soil moisture profiles when digging and colony size differences between the species. These factors promote social living and may help protect individuals from exercise-induced free radical production during digging [15,16,17], and further promote that mole-rats in arid environments are selectively digging during periods when the soil is workable [21,57,66]. Moreover, mole-rats may likely stop digging when at risk of hyperthermia [67].

In contrast to our initial predictions, TAC changes, and the concomitant OSI changes were significantly affected by aridity, which decreased with reduced aridity. A lower OSI would be observed, suggesting efficient ROS scavenging for individuals inhabiting arid environments in the kidneys. Non-enzymatic antioxidants are primarily obtained from food [115,116,117], with others created endogenously in an organism [118,119]. Interestingly, this trend is only observed in the kidneys, which may suggest that the food obtained may selectively enhance antioxidants in a tissue-specific manner [79,120,121] or prioritise kidney antioxidants through inter-organism transports of antioxidants [122,123]. Factors contributing to TAC differences can also be related to the different environments associated with aridity. Previous research has found a greater variety of geophytes available for arid mole-rats [21,49], suggesting that arid individuals have a wider dietary niche as a consequence of increased N and C isotopic values across all tissues [124]. Some geophytes with high antioxidants are only found in arid environments of Steinkopf [125,126]. Furthermore, alternative food sources such as clover and grass were also utilised [124]. As predicted by the arid food-distribution hypothesis, the reduced food density in arid environments may have relaxed the dietary specialization of arid dwelling mole-rats in order for them to adapt and survive in more arid habitats [124]. At the same time, geophytes in arid environments contain significantly more water [21], which may be a pre-emptive measure for plants to combat excessive ROS as a consequence of drought [125,127,128]. This pre-emptive measure to combat drought is also associated with elevated antioxidants in the plants and even their roots, which is likely to occur more regularly in arid environments [125]. It may be that food sources for the common mole-rat in arid environments are richer in antioxidants, compared to more mesic habitats, but this requires further investigation. Since group sizes were not accounted for in the current study, we cannot say with certainty that our data supports the aridity food-distribution hypothesis, however, access to the possibility of a variety of different food sources leading to higher TAC values compared to the semi-arid population suggests that arid animals have sufficient access to food.

The captivity effect on antioxidant activity also needs to be considered. Firstly, food is provided and no longer foraged for, which may decrease the metabolic rate, where a drop in resting metabolic rate was observed after 3 months of acclimation to captivity [58]. The observed acclimation was observed in mole-rats caught only from the most arid region, Steinkopf. The drop in metabolism may affect endogenous antioxidant production [129], likely affecting measured antioxidant levels. Furthermore, glutathione and other antioxidants are rapidly turned over [130] suggesting that observed antioxidant levels may result from captive feeding. Interestingly, if captive feeding was the only source of antioxidants and feeding acclimation occurred, mole-rats from all populations would have demonstrated the same TAC. This suggests that even if dietary acclimation occurred, arid dwelling mole-rats still possess higher non-enzymatic antioxidants in their kidneys and selectively protect them. Other variables which can influence observed levels include the rate of consumption of food by mole-rats which was not controlled for. Furthermore, if additional food consumption took place, the excess antioxidant could result in oxidative damage [131,132], but oxidative damage did not occur, suggesting the mole-rats incorporated the same amount of antioxidants into their system from exogenous sources.

Mammalian oxidative ecology demonstrates that generally, at the basal level, SOD activity is higher in the liver as compared to the kidneys [115,133]. The current study found that kidney SOD activity was slightly higher than that of the liver, and this pattern was consistent regardless of aridity. This is in contrast to other subterranean species which followed the general mammalian trend of higher SOD activity in the liver, such as Brandt’s vole [10] and the Damaraland mole-rat [134]. It is not unusual for kidney SOD activity to be higher than liver SOD activity, as this trend has previously been observed in Wistar rats [135,136], broiler chickens [137] and North American beavers [115]. The common mole-rat may represent one of the species with inherently higher SOD activity in the kidneys as compared to the liver. Alternative ecological explanations for this phenomenon may include hormesis, the process of protection against an oxidative insult resulting in an overall benefit [138,139,140,141]. The kidneys play a critical role in the water balance and retention in mole-rats as they only obtain water from their food resources [62], and the inability of mole-rats to significantly concentrate urine [142], may necessitate protection of kidneys from oxidative insults to maintain optimal kidney function. Additionally, SOD activity levels in the kidneys demonstrated a near significant negative correlation with age, a likely factor in preserving the longevity of common mole-rats. Liver SOD activity also demonstrated a negative correlation, but not to the degree of the kidney. Lastly, an inverse relationship was observed for SOD in relation to MDA for both tissues, but its effect was significant only for the kidneys. Furthermore, this effect was only significant for the arid and semi-arid population and not for the mesic population. This trend being only significant for mole-rats occupying arid and semi-arid habitats suggests prioritised protection of the kidney as compared to the liver in an arid environment, likely due to its involvement in water retention [142,143,144] and possibly its susceptibility to hypoxia [145], a lifestyle associated with subterranean and/or fossorial animals [53,54]. Water is likely still a precious commodity despite its apparent abundance for social mole-rats, and emphasises the importance of kidneys in the common mole-rat and likely other mole-rats for future tissue oxidative status considerations.

One point of note of the current study is that all three sites have experienced aridification, although this effect was most profound in the mesic and arid sites. Aridification can influence vegetation, precipitation and soil properties [6,7,8,9,10], all ecological constraints which explain why mole-rats have become social and increased in group size as predicted by the arid food-distribution hypothesis [21,51]. Despite the mesic site being still considered mesic, this phenomenon highlights the concern about the rate of aridification of this site. It is uncertain whether current colony sizes may have contributed to differences observed in kidney TAC values due to reduced efficiency in group foraging and/or the possible differences in vegetation, and vegetation as a food source and antioxidant content. If the aridity food-distribution hypothesis holds true, if the mesic population does not increase group size with increasing aridity, it may limit the amount of food that is foraged and likely force increased cooperation in the mesic-dwelling social mole-rats. Since TOS and MDA did not change regardless of aridity in each tissue, our results suggest that aridification did not significantly affect oxidative damage at any degree of aridity.

There were some limitations to the current study where future studies may want to direct their focus. Our first concern is the possible acclimation of animals to captivity conditions, which could confound the mechanism behind our observation. As such, future studies may benefit from in-field sampling; however, this requires preserving samples in remote locations. Alternatively, animals may need to be sacrificed sooner to prevent captivity acclimation. Furthermore, the physiological mechanism can be investigated between populations focusing on the kidneys, such as the diuretic hormone vasopressin [3] and more in-depth exogenous antioxidants used by the kidneys, such as targeting glutathione, vitamins C and E, carotenoids etc. [146]. Also, mole-rat food (e.g., geophytes), as suggested, may differ in exogenous antioxidants along an aridity gradient, thus, future studies investigating aridification should collect food items and determine antioxidant content. Lastly, although we observed that the common mole-rat was unaffected by oxidative stress as a consequence of aridification, this observation may only be true for the social species, as aridification may pose a significant risk for solitary mole-rats. Hart et al. [60] found that the arid-dwelling solitary mole-rat species, the Namaqua dune mole-rat (*Bathergus janetta*), is close to local extinction, possibly due to the aridification of their habitat. The Namaqua dune mole-rat and other solitary mole-rats have been observed to possess the lowest plasma TAC when compared to their social counterparts, with the lowest observed for the Namaqua dune mole-rat, the only arid-dwelling solitary mole-rat species (Jacobs unpublish. data). This may suggest that solitary mole-rats may be more adversely affected by aridification and should be considered for future investigation for conservation purposes.

## 5. Conclusions

This study found that wild, social, common mole-rats in arid environments value and selectively protect their kidneys with higher antioxidants. The protection of the kidneys may have longevity consequences, but as it stands, social mole-rats do not suffer oxidative stress from aridification in the liver or kidney. An imbalance between oxidative damage and defence (OSI) can have detrimental effects on a mammal’s health and life span. It is clear that aridity affects this balance, and in light of climate change, it is critical to address the dearth of knowledge regarding the effect of aridity on a mammal’s oxidative ecology.

## Figures and Tables

**Table 1:** The linear best model output for kidney oxidative markers, namely total oxidant status (TOS), non-enzymatic total antioxidant activity (TAC), oxidative stress index (OSI), malondialdehyde (MDA) and superoxide dismutase (SOD), for the common mole-rat (*Cryptomys hottentotus hottentotus*) in response to body mass, age, aridity, sex and the interaction between sex and aridity determined through backwards elimination based on the Akaike information criterion.

Kidney	Variables Kept in Best Model	R^2^	Adjusted R^2^	F (df Regression, df Residual)	*p* (<0.05)
TOS	Aridity + Sex + Aridity*Sex	0.22	0.06	1.34 (5, 24)	0.28
TAC	Aridity + Sex	0.41	0.35	6.13 (3, 26)	0.003 *
OSI	Aridity + Sex	0.32	0.24	4.02 (3, 26)	0.02 *
MDA	No variable	0.13	−0.15	0.46 (7, 22)	0.86
SOD	Age	0.11	0.08	3.53 (1, 28)	0.07

Note: Full linear model before backwards elimination includes body mass, age, aridity, sex and sex*aridity as variables. * indicates significance (*p* ≤ 0.05).

**Table 2:** The linear best model output for liver oxidative markers, namely total oxidant status (TOS), non-enzymatic total antioxidant activity (TAC), oxidative stress index (OSI), malondialdehyde (MDA) and superoxide dismutase (SOD), for the common mole-rat (*Cryptomys hottentotus hottentotus*) in response to body mass, age, aridity, sex and the interaction between sex and aridity determined through backwards elimination based on the Akaike information criterion.

Liver	Variables Kept in Best Model	R^2^	Adjusted R^2^	F (df Regression, df Residual)	*p* (<0.05)
TOS	No variable	0.15	−0.13	0.53 (7, 22)	0.80
TAC	No variable	0.09	−0.20	0.31 (7, 22)	0.94
OSI	No variable	0.09	−0.20	0.32 (7, 22)	0.94
MDA	No variable	0.21	−0.04	0.84 (7, 22)	0.56
SOD	Aridity + Sex + Aridity*Sex	0.29	0.14	1.98 (5, 24)	0.12

Note: Full linear model before backwards elimination includes body mass, age, aridity, sex and sex*aridity as variables. * indicates significance (*p* ≤ 0.05).

## Data Availability

Data is contained within the article or supplementary materials.

## Supplementary Materials

The following supporting information can be downloaded at: https://www.mdpi.com/article/10.3390/antiox11112290/s1, Supplementary S1: The liver and kidney oxidant and antioxidant status difference between tissues; Table S2: The full backwards elimination models of all statistical outputs for total oxidant status (TOS), malondialdehyde (MDA), total antioxidant capacity (TAC), superoxide dismutase (SOD) and oxidative stress index (OSI) for kidney and liver; Table S3: The raw data for common mole-rats *Cryptomys hottentotus* hottentotus, consisting of total oxidant status (TOS), malondialdehyde (MDA), total antioxidant capacity (TAC), superoxide dismutase (SOD), oxidative stress index (OSI), age, body mass, sex and aridity for kidney and liver [147,148,149,150,151,152,153,154,155,156].
