# Supplementary material for: Tissue Oxidative Ecology along an Aridity Gradient in a Mammalian Subterranean Species
Source: Antioxidants (Basel). 2022 Nov 18;11(11):2290. doi: 10.3390/antiox11112290 (PMC9687062; doi:10.3390/antiox11112290)
Supplement: Supplementary file 1 [file antioxidants-11-02290-s001.zip › Supplementary S1. Tissue oxidative status differences.pdf]

Supplementary material

Liver and kidney oxidative status

Due to the vulnerability of the liver and kidney tissue to oxidative stress, these organs typically have increased antioxidant activity [1]. Both tissues demonstrate vulnerability to heat and dehydration-induced oxidative stress [2-6]. Liver and kidney tissues differ in their oxidative status in several aspects; firstly, differences in antioxidant capacity are apparent as glutathione, the main thiol used for antioxidant scavenging [7], is primarily produced and stored in the liver, resulting in the liver being generally higher in non-enzymatic antioxidant activity [8-11]. Enzymatic antioxidants also differ, with superoxide dismutase (SOD) existing in three different isoenzymes, namely: Cu/Zn SOD, MnSOD and FeSOD [12]. The Cu/Zn SOD is the most abundant isoenzyme, these isoenzymes varying not just within but between tissues depending on species, with the general trend being that total liver SOD is higher than kidney total SOD [13]. SOD levels can also vary depending on the stressor present within a tissue and the susceptibility of the tissue to the stressor; for example, heat stress will likely affect the liver more than the kidneys [14]. SOD markers and other antioxidant enzymes are generally affected by age, which can reduce observed enzyme activity levels [15,16]. Oxidative damage is also highly dependent on the rate of free radical production, with the liver and kidney being metabolically active tissues [13,17]. These tissues differ in their mitochondrial respiration rates [18], where respiration rates contribute to radical production as a by-product of respiration [19,20]. In *Rattus norvegicus* rats, the kidneys have a higher respiration rate compared to the liver [18]. This may explain why malondialdehyde (MDA), a marker of lipid damage following circadian variations, was higher in the kidneys compared to the liver [21]. For one mole-rat species where the liver and kidney were investigated, the liver and kidney had similar MDA levels, where MDA was slightly higher in the kidneys in non-breeding individuals [22]. In the same way, total oxidant status (TOS) will also be affected by the rate of free radical production, TOS being a measure of all hydroperoxides present as opposed to the single product of lipid peroxidation, such as MDA [23].

References

1. Limón-Pacheco, J.; Gonsebatt, M.E. The role of antioxidants and antioxidant-related enzymes in protective responses to environmentally induced oxidative stress. *Mutation Research/Genetic Toxicology and Environmental Mutagenesis* **2009**, *674*, 137-147, doi:10.1016/j.mrgentox.2008.09.015.
2. Jacobs, P.J.; Oosthuizen, M.; Mitchell, C.; Blount, J.D.; Bennett, N.C. Heat and dehydration induced oxidative damage and antioxidant defenses following incubator heat stress and a simulated heat wave in wild caught four-striped field mice Rhabdomys dilectus. *PloS one* **2020**, *15*, e0242279, doi:10.1371/journal.pone.0242279.
3. Slimen, I.B.; Najar, T.; Ghram, A.; Dabbebi, H.; Ben Mrad, M.; Abdrabbah, M. Reactive oxygen species, heat stress and oxidative-induced mitochondrial damage. A review. *International Journal of Hyperthermia* **2014**, *30*, 513-523, doi:10.3109/02656736.2014.971446.
4. Habashy, W.S.; Milfort, M.C.; Rekaya, R.; Aggrey, S.E. Cellular antioxidant enzyme activity and biomarkers for oxidative stress are affected by heat stress. *International journal of biometeorology* **2019**, *63*, 1569-1584, doi:10.1007/s00484-019-01769-z.
5. França, M.; Panek, A.; Eleutherio, E. Oxidative stress and its effects during dehydration. *Comparative Biochemistry and Physiology Part A: Molecular & Integrative Physiology* **2007**, *146*, 621-631, doi:10.1016/j.cbpa.2006.02.030.
6. Altan, Ö.; Pabuçcuoğlu, A.; Altan, A.; Konyalioğlu, S.; Bayraktar, H. Effect of heat stress on oxidative stress, lipid peroxidation and some stress parameters in broilers. *British Poultry Science* **2003**, *44*, 545-550, doi:10.1080/00071660310001618334.

7. Balcerczyk, A.; Bartosz, G. Thiols are main determinants of total antioxidant capacity of cellular homogenates. *Free radical research* **2003**, *37*, 537-541, doi:10.1080/1071576031000083189.
8. Bharti, V.K.; Srivastava, R.; Kumar, H.; Bag, S.; Majumdar, A.; Singh, G.; Pandi-Perumal, S.; Brown, G.M. Effects of melatonin and epiphyseal proteins on fluoride-induced adverse changes in antioxidant status of heart, liver, and kidney of rats. *Advances in pharmacological sciences* **2014**, *2014*, doi:10.1155/2014/532969.
9. Lauterburg, B.H.; Adams, J.D.; Mitchell, J.R. Hepatic glutathione homeostasis in the rat: efflux accounts for glutathione turnover. *Hepatology* **1984**, *4*, 586-590, doi:10.1002/hep.1840040402.
10. Hellsten, Y.; Svensson, M.; Sjödin, B.; Smith, S.; Christensen, A.; Richter, E.; Bangsbo, J. Allantoin formation and urate and glutathione exchange in human muscle during submaximal exercise. *Free Radical Biology and Medicine* **2001**, *31*, 1313-1322, doi:10.1016/S0891-5849(01)00631-1.
11. Liu, J.; Yeo, H.C.; Overvik-Douki, E.; Hagen, T.; Doniger, S.J.; Chu, D.W.; Brooks, G.A.; Ames, B.N. Chronically and acutely exercised rats: biomarkers of oxidative stress and endogenous antioxidants. *Journal of Applied Physiology* **2000**, *89*, 21-28, doi:10.1152/jappl.2000.89.1.21.
12. Bowler, C.; Van Camp, W.; Van Montagu, M.; Inzé, D.; Asada, K. Superoxide dismutase in plants. *Crit. Rev. Plant Sci.* **1994**, *13*, 199-218, doi:10.1080/07352689409701914.
13. Marklund, S.L. Extracellular superoxide dismutase and other superoxide dismutase isoenzymes in tissues from nine mammalian species. *Biochemical Journal* **1984**, *222*, 649-655, doi:10.1042/bj2220649.
14. Flanagan, S.; Ryan, A.; Gisolfi, C.; Moseley, P. Tissue-specific HSP70 response in animals undergoing heat stress. *American Journal of Physiology-Regulatory, Integrative and Comparative Physiology* **1995**, *268*, R28-R32, doi:10.1152/ajpregu.1995.268.1.R28.
15. Kasapoglu, M.; Özben, T. Alterations of antioxidant enzymes and oxidative stress markers in aging. *Experimental gerontology* **2001**, *36*, 209-220, doi:10.1016/S0531-5565(00)00198-4.
16. Tiana, L.; Caib, Q.; Wei, H. Alterations of antioxidant enzymes and oxidative damage to macromolecules in different organs of rats during aging. *Free Radical Biology and Medicine* **1998**, *24*, 1477-1484, doi:10.1016/S0891-5849(98)00025-2.
17. Sani, M.; Sebaï, H.; Gadacha, W.; Boughattas, N.A.; Reinberg, A.; Mossadok, B.A. Catalase activity and rhythmic patterns in mouse brain, kidney and liver. *Comparative Biochemistry and Physiology Part B: Biochemistry and Molecular Biology* **2006**, *145*, 331-337, doi:10.1016/j.cbpb.2006.08.005.
18. Hulbert, A.J.; Turner, N.; Hinde, J.; Else, P.; Guderley, H. How might you compare mitochondria from different tissues and different species? *J. Comp. Physiol. B.* **2006**, *176*, 93-105, doi:10.1007/s00360-005-0025-z.
19. Halliwell, B. Biochemistry of oxidative stress. *Biochemical Society Transactions* **2007**, *35*, 1147-1150, doi:10.1042/bst0351147
20. Apel, K.; Hirt, H. Reactive oxygen species: metabolism, oxidative stress, and signaling transduction. *Annu. Rev. Plant Biol.* **2004**, *55*, 373, doi:10.1146/annurev.arplant.55.031903.141701.
21. Sani, M.; Ghanem-Boughanmi, N.; Gadacha, W.; Sebai, H.; Boughattas, N.A.; Reinberg, A.; Ben-Attia, M. Malondialdehyde content and circadian variations in brain, kidney, liver, and plasma of mice. *Chronobiology International* **2007**, *24*, 671-685, doi:10.1080/07420520701535720.
22. Schmidt, C.M.; Blount, J.D.; Bennett, N.C. Reproduction is associated with a tissue-dependent reduction of oxidative stress in eusocial female Damaraland mole-rats (*Fukomys damarensis*). *PloS one* **2014**, *9*, e103286, doi:10.1371/journal.pone.0103286.
23. Erel, O. A new automated colorimetric method for measuring total oxidant status. *Clinical biochemistry* **2005**, *38*, 1103-1111, doi:10.1016/j.clinbiochem.2005.08.008.
